# A qualitative study protocol of ageing carers’ caregiving experiences and their planning for continuation of care for their immediate family members with intellectual disability

**DOI:** 10.1186/s12877-017-0473-9

**Published:** 2017-04-07

**Authors:** Lisa Pau Le Low, Wai Tong Chien, Lai Wah Lam, Kayla Ka Yin Wong

**Affiliations:** 1 School of Health Sciences, Caritas Institute of Higher Education, 18, Chui Ling Street, Shatin, NT Hong Kong; 2 School of Nursing, The Hong Kong Polytechnic University, Hung Hom, Kowloon, Hong Kong; 3 The Nethersole School of Nursing, The Chinese University of Hong Kong, Esther Lee Bldg, Shatin, NT Hong Kong

**Keywords:** Ageing, Community-dwelling, Family caregiving, Intellectual disability, Qualitative research

## Abstract

**Background:**

Understanding the difficulties and needs of the family carers in taking care of a person with ID can facilitate the development of appropriate intervention programmes and services to strengthen their caring capacity and empower them to continue with their caring roles. This study aims to explore ageing family carers’ caregiving experiences and the plans they have to provide care for themselves and their ageing children with mild or moderate intellectual disability (ID).

**Method:**

A constructivist grounded theory will be used to interview around 60 carers who have a family member with mild or moderate ID and attending sheltered workshops in Hong Kong. Constant comparative analysis methods will be used for data analysis.

**Discussion:**

The theory will capture family caregiving experiences and the processes of carers in addressing caregiving needs, support received and plans to continue to provide care for themselves and their relatives with ID in their later life. New insights into the emerging issues, needs and plights of family caregivers will be provided to inform the policies and practices of improving the preparation for the ageing process of the persons with ID, and to better support the ageing carers. The theoretical framework that will be generated will be highly practical and useful in generating knowledge about factors that influence the caregiving processes; and, tracking the caregiving journey at different time-points to clearly delineate areas to implement practice changes. In this way, the theoretical framework will be highly useful in guiding timely and appropriate interventions to target at the actual needs of family carers as they themselves are ageing and will need to continue to take care of their family members with ID in the community.

## Background

Family caregiving of their loved ones can be a satisfying and joyful experience. Despite these satisfying outcomes, a main concern has been about the family carer’s health and well-being in general. Family caregiving is known to be a formidable task that is highly emotional and burdensome, and requires timeless commitment and efforts [1–5]. Indeed, the issue concerning the health and safety of carers owing to the caregiving demands that put them at risks for injury and adverse events has already been highlighted by Reinhard et al.’s work [6] on supporting family carers in providing care. Another key caring issue they highlighted is on the need to help carers learn to be competent people to better protect the care recipients from harm [6]. These issues are especially true for the family carers of persons with intellectual disability (ID) who may be providing care and support of varying scope, intensity and duration. Regardless of whether the person with ID is a young child or an adult, or has varying grades of severity - mild, moderate or severe ID - family carers will always have concerns about the person’s caring needs, and sourcing supportive services and resources that can help them relieve their concerns so that they can continue to manage their loved one’s future welfare and well-being as the ID sufferer approaches old age.

With the awareness of the imminent increasing number of ageing persons with ID, the World Health Organization (WHO) and some international organizations have put emphasis on promoting the general health status of this group of people, and explored conditions that support their longevity [7]. Specific to the carers of these persons, there is limited information about the transitions in the caring process in which carers pass through over time in order to meet the range of changing caring needs of the ID sufferers. Therefore, this study will explore the crux and extent of the caregiving situation. This will serve to collate the current caregiving experiences and the support received directly from the carers concerned, and their plans for future care and the well-being of ID sufferers as they are approaching old age.

Intellectual disability is classified under mental disorders by the WHO. ID is defined as a person who has ‘significantly reduced ability to understand new or complex information and to learn to apply these new skills (i.e. impaired intelligence). This results in a reduced ability to cope independently (i.e. impaired social functioning), and begins before adulthood, with a lasting effect on development’ ([8], p.3). A person is diagnosed with ID by assessing three areas: (1) level of intelligence using an Intelligent Quotient (IQ) test, (2) level of independent functioning using a validated measure or clinical observation, and (3) evidence that these impairments were present before aged 18 years [8]. Based on this assessment, local policies have been enacted to protect persons with ID in the areas of education, employment and rehabilitation.

Comparatively, while the prevalence rate of the number of people with ID has been 0.05–1.55% [9], statistics for Asia is at 0.06–1.3% [10]. Indeed, over the past few years in Hong Kong, there has been an incremental increase in the number of persons with ID from 2008 to 2014 as well. According to the government statistics in 2008, of 269,500 people with disabilities in Hong Kong, 62,000 to 87,000 were persons with ID, giving rise to a prevalence rate of 23 to 32% [11]. By 2014, there were 578,600 persons with disabilities (8.1% of the total Hong Kong population). The numbers have increased from 71,000 to 101,000 persons with ID [12]. Although it is acknowledged that people with ID are living and surviving longer and that quite a number are also reaching old age, it has been difficult to obtain statistics to support this claim. This has partly been attributed to the problems encountered in the under-reporting of persons with ID as mentioned in the government reports [11, 12]. Indeed, a recent news report in Hong Kong [13] also highlighted the inconclusive evidence on how to define an ageing person with ID and their average life expectancy. Without a clear consensus on this issue, instilling coordinated approaches to address the problems of ageing ID sufferers could be difficult. Despite this, a government paper [14] has outlined the support provided and measures taken in response to the ageing of persons with ID particularly on the support services, and issues of manpower, equipment and services in the service units.

The impetus to undertake this study is also timely supported by a recent alarming newspaper report entitled a ‘huge study on the disabled revealed as government accused of ignoring ageing problems’ [13]. It stated that more than 11,000 people with ID who were surveyed in the past year mainly relied on the care provided by their elderly parents. Those surveyed were currently using day centres, care homes and employee support services. Indeed, the plights and conditions of the carers who are also ageing themselves, and their high concerns for the future and continuing welfare of the soon-to-be old family persons whom they are looking after were clearly evident. This outcry was a demand for more help and attention to be given to address the multiple needs of ID sufferers and to provide longer term support such as government subsidized nursing homes or infirmary care units.

Over the past decade, scholarly work has shed some understanding of the caring issues faced by family carers of ID persons. For carers, Mak and Ho’s work [15] on caregiving perceptions of 212 Chinese mothers of children with ID in a non-governmental organization was an attempt to consider the coping style and the experience that could be unique to the Chinese culture. Relationship-focused coping strategies were found to be more suitable for understanding Chinese carers’ perceptions of caregiving stress, burden and family support as opposed to problem-focused and emotion-focused coping strategies. The follow-up work by Lin [16] was a typical example of local work that surveyed the high stress level in caregiving and its impacts on the psychological well-being of 54 parents rearing children with severe ID. The work was able to extend an understanding of perceived stress and coping strategies with their subjectively perceived quality of life. Of the three major stressors identified as the caregiving tasks (such as bathing, toileting, shopping, lifting and physical handling), future planning and child’s health, internal coping strategies were adopted to manage these stressors and parents were less likely to seek help from others. There are also studies conducted to examine stressors, caring burden and coping strategies of parents immediately after the birth and acceptance of a child with ID [17, 18]. These studies suggest areas for further work by exploring and describing in greater depth the nature and impacts of the stressors on family carers’ ability to handle the daily caregiving tasks. Additionally, the kind of support and assistance that these carers would expect to support their needs warrant greater attention, especially when they tend to decline help from others, particularly health care professionals.

For the ID sufferers, some qualitative studies conducted have shed understanding on the provision of life skills training, guidance and employment support programmes so that they can lead meaningful lives, and minimize the strain on their families [19]. Findings from a study on vocational aspiration and the level of assistance needed in decision-making of 23 persons with ID in sheltered workshops indicated that they were motivated to have employment, but they had limited self-determination in the workplace [20]. Another study found that the younger persons with ID could have a higher self-concept than that of a comparison group of people without disabilities [21]. Of importance to them were family self, social self and achievement in school and work. Partnership with the family was important in facilitating them to develop positive self-concepts and achieve community integration.

More recently, Li et al. [22] examined the difficulty and burden experienced by family carers of persons with ID. Physical caring was perceived to be significantly more challenging than social caring and daily caring. Similarly, time-dependence burden was more severe than developmental, physical, social and emotional burden. Although the study categorized the types of caring difficulty and caring burden in terms of its importance and severity, there was no information about the transit points along the caregiving journey that could be linked to the different types of caring challenges faced by the carers. An exploration of each categorization using the actual experiences as told by the family members may be more meaningful in planning strategies and making suggestions to target at different time-points of the caregiving journey.

Despite the growing concerns of life satisfaction and health needs of family carers of persons with ID, there is a dearth of studies that uses a comprehensive approach to exploring the range of caregiving experiences by allowing the carers to truly express such experiences using in-depth interviews. Indeed, this study will produce a theoretical framework to capture these experiences in the caring process that can be best understood by studying it over time [23]. To date, studies conducted in Hong Kong have not adopted this approach in capturing the nature of caring needs, responses to address those needs, and to trace the plans for the future care of ID sufferers in their later life. A better understanding of the impact of the family carers needs and concerns over time would allow interventions to reflect changes over time.

The objectives of this study are to:

1. To explore family carers’ perspectives of current caring needs and types of care needed by the person with ID;
2. To examine the extent to which the current level of support has met family carers’ expectations and needs, and the caring needs of the person with ID;
3. To examine the plans family carers have for continuing the care of the person with ID as carers themselves are getting older;
4. To explore how family carers plan to achieve/implement their plans for continuing care of the person with ID person from now until later life.
5. To develop a theoretical framework that captures the family carers’ experiences and processes of planning to meet the current and continuing care needs of community-dwelling persons with ID.

## Method

### Study design

A constructivist grounded theory (GT) design will be used [23] to collect the data from family carers of people with ID using semi-structured interviews. This design posits that knowledge is constructed, modified and tested in the light of new experiences [24]. Knowledge is co-created through the reciprocal relationship that is formed between the researcher and the participants as they work towards exploring an interpretative understanding of the participants’ experiences and acknowledging that they create multiple meanings about the phenomenon [23]. Therefore, when new, divergent or conflicting constructed views and experiences are examined, consensus is reached to describe the patterns of meaning and interrelationships about the phenomenon [25] which will be included into the emerging theory.

### Settings and participants

Three conveniently-selected sheltered workshops to provide services to persons with mild to moderate grades of ID will be selected. According to the Hong Kong Social Welfare Department [26], these sheltered workshops serve to provide persons with disabilities who are not able to enter into open employment with appropriate vocational training in a specially designed environment in order to help them develop their social and economic potential to the fullest extent. It also aims to enhance their working capacity in order that they can move on to supported or open employment wherever possible. These services are targeted at persons with disabilities who are aged 15 years and above and have basic self-care and ability to work. Services provided include training and activities to extend their potential, work habits and various kinds of social and daily living skills, and structured programmes to meet their developmental and social needs.

Purposive sampling will be used in this study to identify those participants who are most likely to provide rich information about the experiences of the phenomena under study. These persons are carers who have an immediate family member with mild (ID 50–69) or moderate (25–49) grades of ID. This can be useful to examine a wide range of family caring experiences for mild and moderate ID persons who may be able to demonstrate a continuum of care needs and support/resources they are currently using. The selection criteria for family members include: (1) primary carer who is aged 50+ years and takes care of the welfare of the person with ID, (2) currently using the services provided by the sheltered workshops, (3) immediate family members (e.g. parents and siblings), and (4) living in Hong Kong with contact information provided. Following the purposive sampling of participants, theoretical sampling will be used to enrich and clarify the important data/information by sampling for people, circumstances, activities and incidents as guided by the codes and categories that are being developed by the emerging theory. More data will be collected to check out events, activities and issues from the subsequent interviews, with the aim to continue theoretical sampling until the full range and variation within a category is fully developed [27]. Therefore, this study will target at sampling about 20 family carers per site and hopefully 60 family carers will be needed to reach data saturation.

### Ethical considerations

Ethical approval to conduct the study was given by the Ethics and Research Committee (Project no.: HRE150112). Each family member will be given an information sheet about the invitation to participate in an interview that will last for about one hour and will be tape-recorded with their agreement. Participants are required to sign a consent form to indicate their willingness to participate. Given the sensitivity associated with the unravelling of emotions surrounding the upbringing and caring issues of the persons with ID, the research team is mindful of this potentially vulnerable group of family carers. Voluntary participation and the right to ask any questions and to decline participation at any time will be emphasized during the data collection.

### Data collection

The timeframe for this study will be 24 months from 1 January 2016 to 31 December 2018. For each site, the research assistant (RA) will conduct concurrent data collection and analysis. For the first study site, five months is planned to complete 20 family interviews. For the other two study sites, data will be collected concurrently and it is estimated that around seven months will be needed to complete 40 family interviews. After ethical approval is obtained, permission to obtain access to the study sites will be gained. The RA will consult workshop staff regarding the grades of ID of the employees and then recruit the respective family members for the pilot and main study. The first author will conduct a few interviews with the RA initially until the RA shows competence and satisfactorily interview skills. In order to ensure the quality of collected data, it is planned that 1–2 in-depth interviews will be conducted per day, depending on the availability of family members. Interviews will last for 45–60 min. The main interview schedule is as follows:

1. Describe the types of care you have provided to the family member with ID?
2. Over the past years, how have you provided the care to ensure that the needs of the person with ID have been met?
3. What do you see as the current caring needs required by the person with ID?
4. Describe the types of support you have received and would expect to help take care of yourself?
5. Describe the types of support you have received and would expect to help take care of your family member with ID?
6. Based on the family member’s current care needs, what concerns do you have about his/her future care?
7. What plans do you have to continue to provide care for him/her as you are approaching old age?
8. What plans do you have to achieve/implement your plans for continuing care of your family member with ID from now until later life?

As the interviews proceed, questions are adapted when the focus, depth and scope of the phenomenon emerge following constant comparative analysis methods. The participants will be interviewed once and will be tape-recorded using a digital voice recorder to ensure that the interview content is accurately captured. After the interviews, field notes will be kept to jot down issues that cannot be audiotaped such as the participants’ gestures or facial expressions.

### Data analysis

Data analysis will be conducted at the same time as data collection as far as possible. The goal is to use the emerging analysis to shape subsequent data collection procedures, and to check participants’ understanding of emerging theoretical categories in order to build the eventual theoretical framework. Indeed, after the interviews have been completed all interview recordings will be transcribed verbatim. This requires checking through each transcript against the taped recordings to ensure accuracy of the content and that the translation of terms and/or words is consistently used throughout. The data will be analyzed using the constant comparative analysis methods. This will generate the concepts that will be used to develop the theory through an inductive process of defining, categorizing, comparing data, and explaining and seeking relationships in the data [23]. The investigators will ensure that a systematic and logical approach will be used to fully describe the data, and then to ensure that all the variations in the data will be compared in order to account for the related properties in each category that emerges. A qualitative data analysis software known as ‘MAXQDA The Art of Text Analysis’ will be used by the investigators and RA to code, organize and manage the data to facilitate data interpretations.

## Discussion

This study will increase our understanding of the difficulties and needs of the family carers in taking care of an ageing adult with ID by facilitating the development of appropriate intervention programmes and services to strengthen their caring capacity and empower them to continue with their caring roles.

The theoretical framework that will be generated from this study will be highly practical and useful in generating knowledge about factors that influence the caregiving processes, and tracking the caregiving journey at different time-points so that areas to implement practice changes can be clearly delineated. In this way, the theoretical framework is highly useful in guiding timely and appropriate interventions and project innovations to address the actual needs of family carers as they themselves are ageing and will need to continue to take care of their family members with ID in the community. Therefore, after the identification of the carers’ caring process for persons with ID, various interventions can be planned to address the changing needs of the carers and also the persons with ID at different points in the trajectory of the caring continuum.

With better support, carers can continue their care for the community-dwelling persons with mild or moderate ID for a longer period of time. This will not only benefit the persons with ID and their carers but also relieves the heavy load of the healthcare system in the provision of institutionalized care. In addition, the findings of this study can provide important information about the psychosocial and service support and needs for family carers of home- or community-residing persons with ID, and for the persons with ID. The needs and plans for caregiving established in this research can inform the current situation and future development of community support services and care policy of family-centred care for persons with ID.

The focus on only those with mild or moderate ID who are attending the sheltered workshops and the exclusion of family caregiving of those who have severe ID will be the limitations of this study. Therefore, the emerging theory may not be transferrable to those with severe ID or those who could not be employed by the sheltered workshops. Indeed, as the participants are interviewed once, identification of subtle changes in the caring needs of individual careers and the person with ID they look after have to be based on the participants’ ability to recall incidents and events that have happened at different points of time. More data sources and longitudinal data collection method can be considered to enrich the data and examine the changes in their health needs over time.

Planning for the continuation of care for an adult child with ID requires good physical and psychological health. For the ageing carers, their future health and the health of the family member they care for is full of uncertainties. Indeed, the health of either party can deteriorate sharply, which may have direct impact on the carers’ plan for continuation of care. It is possible that the participants may express their inability to anticipate their future plan for continuation of care or their inability to take care of their family member in the future and their plan is to seek social service for the family member. Therefore, various perspectives should be explored so that the data will have the potential to inform policies and practices about supporting families to minimize caregiver burden, and to give the individuals with intellectual disabilities a chance to live in the own communities.

## Abbreviations

GT: Grounded theory
ID: Intellectual disability
IQ: Intelligent quotient
RA: Research assistant
WHO: World Health Organization

## References

[1] Innes A, McCabe L, Watchman K (2012). Caring for older people with an intellectual disability: a systematic review. Maturitas.

[2] Low LPL, Fan KP. Discharging older patients from convalescent hospitals: Information needs of family members to inform the development of eLIP. Proceedings of the 40th Annual Conference of British Society of Gerontology; 2011. p.203.

[3] Low LPL, Wong MH, Ling CF, Fan KP. Up against a challenge of providing pre-discharge resources for family carers of older patients: The process of developing a user-friendly eLIP website. Proceedings of the 21st Nordic Congress of Gerontology; 2012. p.127.

[4] Low LPL, Lam LW. Making everyday decisions in residential care homes: Decision-making potential and challenges faced by family members of elders with mild to moderate dementia. Proceedings of the 16th Asia Pacific Regional Conference of Alzheimer’s Disease International; 2013. p.24

[5] Low LPL. Accessing information before the point of need - A CADENZA initiative to digitalize pre-discharge planning for older patients and their family members. Proceedings of the 20th IAGG World Congress of Gerontology and Geriatrics, 2013. p.240.

[6] Reinhard SC, Given B, Petlick NH, Bemis A, Hughes RG (2008). Supporting family caregivers in providing care. Patient safety and quality: an evidence-based handbook for nurses.

[7] World Health Organization (2000). Ageing and intellectual disabilities – improving longevity and promoting healthy ageing: summative report.

[8] World Health Organization (2010). European declaration on the health of children and young people with intellectual disabilities and their families.

[9] Mckenzie K, Milton M, Smith G, Ouellette-Kuntz H (2016). Systematic review of the prevalence and incidence of intellectual disabilities: current trends and issues. Current Developmental Disorders Reports.

[10] Jeevanandam L (2009). Perspectives of intellectual disability in Asia: epidemiology, policy and services for children and adults. Current Opinion in Psychiatry.

[11] Census & Statistics Department (2008). Special topics report no. 48. Persons with disabilities and chronic diseases.

[12] Census & Statistics Department (2014). Special topics report no. 62. Persons with disabilities and chronic diseases.

[13] Ngo J (2015). Huge study on disabled revealed as government accused of ignoring ageing problems.

[14] Hong Kong Government. Ageing of persons with intellectual disabilities. LC Paper No. CB(2)493/13–14(01); 2013. http://www.legco.gov.hk/yr13-14/english/panels/ltcp/papers/ltcp1216cb2-493-1-e.pdf

[15] Mak WWS, Ho SMG (2007). Caregiving perceptions of Chinese mothers of children with intellectual disability in Hong Kong. J Appl Res Intellect Disabil.

[16] Lin LYA (2009). Parents of children with severe intellectual disabilities in Hong Kong: relationship among their perceived stress, coping strategies and subjective quality of life.

[17] Lam LW, Mackenzie AE (2002). Coping with a child with down syndrome: the experiences of mothers in Hong Kong. Qual Health Res.

[18] Kwok SYCL, Leung CLK, Wong DFK (2014). Marital satisfaction of Chinese mothers of children with autism and intellectual disabilities in Hong Kong. J Intellect Disabil Res.

[19] Bai X, Ho DW, Fung K, Tang L, He M, Young KW, Ho F, Kwok T (2014). Effectiveness of a life story work program on older adults with intellectual disabilities. Clinical Intervention Aging.

[20] Li EP (1998). Vocational aspirations of sheltered workshop workers with intellectual disability in Hong Kong. Journal of Intellectual Disability.

[21] Li PYE, Tam SFA, Man WKD (2006). Exploring the self-concepts of persons with intellectual disabilities. J Intellect Disabil.

[22] Li WO, Fan TW, Yu CKC (2012). Difficulty and burden experienced by principal family caregivers of people with intellectual disabilities in Hong Kong. Asia Pacific Journal of Counselling and Psychotherapy.

[23] Charmaz K (2006). Constructing grounded theory: a practical guide through qualitative analysis.

[24] Schwartz SH (1999). A theory of cultural values and some implications for work. Applied Psychology: An International Review.

[25] Guba EG, Lincoln YS, Denzin NK, Lincoln YS (2005). Paradigmatic controversies, contradictions and emerging confluences. Handbook of qualitative research.

[26] Hong Kong Social Welfare Department. Services for mentally handicapped persons; 2015. http://www.swd.gov.hk/doc/rehab/SW_Eng_072014.pdf

[27] Polit DF, Beck CT (2017). Nursing research: generating and assessing evidence for nursing practice.

